# How I Treat: Infections and inborn errors of immunity—Prevention, diagnosis, and treatment

**DOI:** 10.70962/jhi.20250137

**Published:** 2025-12-16

**Authors:** Alexandra F. Freeman, Beth K. Thielen, Tamara C. Pozos

**Affiliations:** 1 Laboratory of Clinical Immunology and Microbiology, National Institute of Allergy and Infectious Diseases, National Institutes of Health, Bethesda, MD, USA; 2 Division of Pediatric Infectious Diseases, Department of Pediatrics, University of Minnesota Medical School, Minneapolis, MN, USA; 3 Department of Clinical Immunology, Children’s Minnesota, Department of Pediatrics, University of Minnesota Medical School, Minneapolis, MD, USA

## Abstract

Infections are a significant cause of morbidity and mortality in inborn errors of immunity (IEI). Prevention of infections relies on knowledge of the specific pathogen susceptibility of the IEI to then consider antimicrobial prophylaxis, immune modulation, and/or environmental measures. In addition, complications of IEI include bronchiectasis, which can lead to more infections due to disruption of mucociliary clearance; suppression of chronic infections and airway clearance measures are important parts of bronchiectasis management. We discuss some infections that are challenging to diagnose for patients with distinct IEI and how partnership with the microbiology laboratory can lead to more prompt diagnosis and specific management. Finally, the increasing ability to identify the molecular basis of IEI facilitates precise immunomodulation, which can attenuate the infection burden of these diseases yet can also add unique infection risks depending on the specific therapy.

## Teaser

Freeman et al. provide practical advice on prevention, diagnosis, and management of infections in inborn errors of immunity as well as management of common complications such as bronchiectasis.

## Introduction

Inborn errors of immunity (IEI) are characterized by varying levels of infection susceptibility, either related to their specific immune defect or, in some cases, related to the immune modulation used to treat immune dysregulation. The best approaches to prevent infections and associated complications, such as bronchiectasis, are of great concern amongst health care professionals and patients. However, due to the rarity of many IEI, controlled studies for the best approaches to infection prevention are limited, and practices are often extrapolated from guidelines from other immune deficient states, such as HIV. Practices also vary from center to center based on past patient experiences.

Although we cannot provide guidance for the over 500 IEI as well as many genetically undefined immunodysregulated states (1), we will review some studies focused on infection prevention in specific IEI and some of our practices for preventing infections in people with specific IEI. In addition, we will provide some pearls around identification and management of some of the trickier infections seen in IEI and IEI mimickers.

## Antimicrobial prophylaxis and immunoglobulin replacement (Tables 1 and 2)

Because of the rare nature of most IEI, randomized placebo-controlled studies are largely lacking. We will review briefly some of the studies and general approaches.

**Table 1.** Antimicrobial prevention in IEI

IEI/Disease process	Antimicrobial regimens	Comments
SCID	TMP/SMX for PJP prophylaxis starting at 1 mo; second line agents include pentamidine, atovaquone, and dapsone Fluconazole for *Candida* prophylaxis Antivirals dependent on risks (2) RSV monoclonal antibody seasonally IgRT within a few weeks of life, trough levels >800 mg/dl	Monitor bilirubin and blood counts on TMP/SMX Folinic acid to minimize cytopenias Monitor liver function tests on fluconazole Avoid breastfeeding if mother CMV IgG positive Irradiated blood products Avoid live vaccines (rotavirus, MMR, varicella, and BCG)
CGD	TMP/SMX twice daily for bacterial prophylaxis Itraconazole for fungal prophylaxis; other mold-active triazoles as alternative include posaconazole Consideration of IFNγ	Remember corticosteroid/azole interaction Environmental considerations to minimize mold/water exposures Diagnosis of lung infections preferably with biopsy Some infections (i.e., *Nocardia* pneumonias, *S. aureus* liver abscess) have better response if corticosteroids added to antimicrobials
HIES	TMP/SMX twice daily for bacterial prophylaxis (consider alternatives such as doxycycline if resistant organisms) Fluconazole for frequent *Candida* infections and in *Coccidioides* endemic regions Itraconazole if pneumatocele is present, posaconazole if chronic mold infection Consider IgRT	Avoid lung surgeries and lung biopsies if possible due to risk of prolonged bronchopleural fistulae Consider dupilumab for eczematoid dermatitis; can also reduce skin infections Antiseptics (dilute bleach baths, chlorhexidine) to minimize *S. aureus* skin colonization Low threshold to look for infection as systemic signs of inflammation are diminished
XLA	IgRT with optimization of trough Consider azithromycin Influenza and SARS CoV2 vaccination can maximize responses through T cell–dependent mechanisms	Low threshold to consider *Campylobacter*/*Helicobacter* with persistent fevers, lower extremity erythema/bruise-like and boggy skin changes Low threshold to look for chronic enterovirus with CNS symptoms Consider Aichi virus with nephritis, hepatitis
Bronchiectasis	Consider azithromycin after sputum cultures for *Mycobacteria* Airway clearance techniques Consider of inhaled antimicrobials	Check EKG for QTc with addition of azithromycin and consider hearing screen Periodic sputum cultures to be aware of colonization and optimal antimicrobials if needed Caution with inhaled tobramycin if renal insufficiency
MSMD	Prophylaxis with azithromycin after NTM infections treated Consideration of IFNγ for treatment in addition to antimicrobials with certain defects (IL12RB1 deficiency, AD IFNγR1 deficiency, NEMO, and IkBα)	Avoid BCG vaccination For complete IFNγR deficiencies, after control of NTM with combination antimicrobials, HSCT is indicated Some risk of endemic mycoses, therefore minimize environmental exposures and in *Coccidioides* endemic regions consider prophylactic azoles
Perioperative	Consideration of *S. aureus* therapies perioperatively with longer course if hardware placed (7–10 days)	*S. aureus* decolonization with nasal mupirocin, antiseptics
Women’s health	Many restrictions of antimicrobials during pregnancy—consider azithromycin for prophylaxis and topical antifungals if needed	Close communication with Obstetrician and IEI practitioner

**Table 2.** Dosages and dosing schedules of common antimicrobials in IEI

Antimicrobial	Dosage	Comments
TMP/SMX	5 mg/kg/day TMP component, divided twice daily; max dose 160 mg TMP 5 mg/kg TMP component divided twice daily 3 days/wk	Twice daily dosing for bacterial prophylaxis in CGD, HIES; consider for PADs 3 days/wk dosing for PJP prevention in SCID, athymia, CID at risk (e.g., Hyper IgM syndrome) Monitor for cytopenias; Folinic acid can minimize risk Consider atovaquone or pentamidine as alternatives for PJP prevention
Azithromycin	5 mg/kg/day; max dose of 250 mg daily or 5 mg/kg/day or 500 mg 3 days/wk	Check EKG for QTc interval and consider hearing screen Monitor for resistance with long-term use
Penicillin	125 mg twice daily <5 years old 250 mg twice daily 5 years and older	Asplenia, terminal complement defects
Amoxicillin	40 mg/kg/day divided twice daily; max dose 500 mg	Mild hypogammaglobulinemia as an alternative to IgRT
Fluconazole	5–6 mg/kg/day; max 200 mg daily	Consider drug interactions Monitor transaminases Prophylaxis against yeast; no filamentous mold (e.g., *Aspergillus*) activity
Itraconazole	5–10 mg/kg/day; max 100 mg (<50 kg) or 200 mg (>50 kg)	Consider drug interactions and monitoring drug levels Monitor transaminases
Posaconazole	300 mg tablets daily for adults and children >40 kg 6 mg/kg/day of tablets for children <40 kg	Tablet dosing differs from suspension dosing Consider drug interactions Check trough level and transaminases 1–2 wk after initiation Check EKG for QTc
Acyclovir	80 mg/kg/day divided twice daily or TID 400 mg twice daily adult dosing for HSV/Varicella/Zoster (VZV) suppression	Encourage hydration and monitor renal function Can use in patients with SCID if at risk for HSV Do not use if on valganciclovir Consider acyclovir or valacyclovir with JAK inhibition
Valacyclovir	500–1,000 mg daily for adults 20 mg/kg/day divided twice daily to 500 mg twice daily max for pediatrics	Do not use if on valganciclovir Consider acyclovir or valacyclovir with JAK inhibition
Valganciclovir	10–20 mg/kg daily, max 900 mg/day	Monitor for cytopenias and elevated transaminases For SCID with suspicion of CMV exposure or CMV disease

### Severe combined immune deficiency (SCID)

Due to the severity of SCID with high mortality rates in infancy without curative immune reconstitution through hematopoietic stem cell transplantation (HSCT), gene therapy, or enzyme replacement, guidelines for diagnosis and treatment have been generated through expert consortia (e.g., primary immune deficiency treatment consortium) (2). Entering HSCT without infection significantly improves outcomes; therefore, prevention, detection, and treatment of infections are critical (3). The key infections to prevent or to detect early and treat are (1) *Pneumocystis jirovecii* pneumonia (PJP) with trimethoprim/sulfamethoxazole (TMP/SMX) as typical prophylaxis starting at 1 mo of age, (2) fungal infections (e.g., *Candida*) with fluconazole commonly used, (3) herpes viruses, including cytomegalovirus (CMV), with a minority of centers using empiric valganciclovir without evidence or concern of infection. Monoclonal antibodies to prevent respiratory syncytial virus (RSV) infections (e.g., nirsevimab) are appropriate during RSV season. Prompt diagnosis of bacterial infections such as pneumonia and common respiratory viruses is also important given the extreme immunosuppression. Immunoglobulin (Ig) replacement therapy (IgRT) is also typically recommended for all infants with SCID within the first weeks of life. While there is reported variability in infection prevention for SCID between centers, we support the recommendations by Dorsey et al. for choice of antimicrobial agents and dosing (2).

Certain exposures should also be avoided for SCID; for example, transfusions should be limited to irradiated blood products (2). There is controversy about the risk/benefit ratio of breastfeeding for infants with SCID; to our knowledge, the most recent study addressing this was in 2019, where Kelty et al. showed CMV transmission of 5% to infants through breastfeeding (4). Although this risk is low and we recognize the established benefits of breastfeeding, in our practice we recommend against breastfeeding if the mother’s serostatus is unknown or positive. In addition, we do not use valganciclovir as prophylaxis unless there is infant CMV infection. General environmental isolation practices vary between centers (2); if the infant is isolated in an outpatient setting, efforts should be made to minimize contact with school aged children, people with active herpes simplex virus (HSV)-1 or recent vaccination with live vaccines, and home environments under construction.

### Chronic granulomatous disease (CGD)

The benefit of antibiotic prophylaxis with TMP/SMX for CGD has been standard of care for decades, and retrospective data showed a decrease in bacterial infections by about 50% (5). TMP/SMX provides coverage for the most common bacterial pathogens in CGD, including *Staphylococcus aureus*, *Burkholderia cepacia, Serratia marcescens*, and *Nocardia* species. The first placebo-controlled antimicrobial prophylaxis study in IEI was a crossover study of itraconazole for patients with CGD (6). A notable decrease in fungal infections was seen, and itraconazole with TMP/SMX became standard. Itraconazole is available in a liquid suspension and capsules; the suspension has better absorption but can be associated with reduced compliance, and so capsules are encouraged when feasible. Prior to the routine use of antifungal prophylaxis in CGD, a randomized placebo-controlled study showed that the addition of interferon-gamma (IFNγ) decreased serious infections by about two thirds, and so many centers use IFNγ in addition to TMP/SMX and itraconazole (7). We suggest the review by Thomsen et al. for additional information regarding CGD management (8).

### Hyper IgE syndrome (HIES)

There are no randomized placebo-controlled studies for HIES; however, retrospective and cohort studies show that most patients with STAT3-HIES (Job’s syndrome) are given antibiotic prophylaxis, largely with TMP/SMX to prevent *S. aureus* lung and skin infections (9, 10). Minimizing *S. aureus* colonization of the skin can greatly improve the eczematoid dermatitis that these patients have; however, resistance to TMP/SMX and other antibiotics can occur. Therefore, using antiseptics to reduce the *S. aureus* load on the skin is prudent, such as with dilute bleach baths (typically ½ cup to a tub for 15 min 3 days/wk, followed by moisturizer), swimming in chlorinated pools, and/or bathing with chlorhexidine (10). In recent years, dupilumab has been increasingly used successfully to treat the dermatitis in HIES, making antiseptics less necessary (11). Azithromycin is used frequently for those with bronchiectasis as additional prophylaxis (discussed below). IgRT has also been shown retrospectively to decrease bacterial pneumonias (9) and is given typically for those with breakthrough infections despite antibiotic prophylaxis and/or those with poor specific antibody responses.

We recommend antifungal prophylaxis based on individualized risks: (1) all patients residing or traveling to *Coccidioides* endemic regions should be on an azole antifungal due to the risk of *Coccidioides* meningitis (12); (2) fluconazole chronic therapy can be considered for those with frequent *Candida* nail or oral infections; antifungal resistance can occur, but is not thought to be more common for those on chronic vs. intermittent therapy (13); (3) itraconazole prophylaxis is suggested for those with pneumatoceles to minimize the risk of aspergillomas. Finally, chronic mold therapy, such as posaconazole, is typically provided for those with history of pulmonary mold infections. We also recommend anti-mold prophylaxis for patients with increased exposures, such as living/working on a farm.

### Primary antibody deficiencies (PADs)

The standard of care for those with significant antibody deficiencies, such as X-linked agammaglobulinemia (XLA) and chronic variable immunodeficiency (CVID), is IgRT through intravenous (IVIG) or subcutaneous delivery. Various combined immunodeficiencies (CIDs), such as activated phosphoinositide 3-kinase delta syndrome or DOCK8 deficiency, can also confer a component of humoral immunodeficiency and patients benefit from IgRT. In one report of patients receiving IVIG, the trough level was correlated with the risk of pneumonia, with five times more pneumonias seen with an IgG trough of 500 mg/dl compared to a trough of 1,000 mg/dl (14). However, if infections continue to occur despite IgRT, or if there is significant pulmonary disease, antibiotics are typically added to the IgRT (15).

A key placebo-controlled study for antibiotic prophylaxis in PADs and chronic lung disease studied azithromycin prophylaxis (16). Azithromycin in this study was dosed at 250 mg 3 days/wk and decreased the frequency of bronchiectasis exacerbations needing antibiotics or hospitalizations, leading to the frequent use in this setting. Recommendations for dosing and frequency of azithromycin vary from 250 to 500 mg (for pediatrics, 5 mg/kg/dose) 3 days weekly to 250 mg daily. We typically use 250 mg daily (5 mg/kg in children) for better protection against some other infections to which these patients are susceptible, such as *Mycoplasma* and, in the case of XLA, *Helicobacter* and *Campylobacter* (discussed more below). We suggest doing an electrocardiogram (EKG) prior to initiation of long-term azithromycin to check for prolonged QTc, especially as this risk is increased if other QTc prolonging medications are added (e.g., quinolones and azoles). Long-term azithromycin can also be associated with hearing loss, especially when given at higher doses (e.g., 500 mg daily), and counseling about this risk and a baseline hearing exam may be considered. Of note, if bronchiectasis is present, there is a risk of nontuberculous mycobacterial (NTM) pulmonary disease (see below).

For mild hypogammaglobulinemia and/or specific antibody deficiency, it is possible to consider daily antibiotic prophylaxis with azithromycin, amoxicillin, or TMP/SMX. In a randomized crossover study, prophylactic antibiotics had comparable efficacy to IgRT in patients with mild humoral defects (17). For these patients, IgRT can then be considered as a next step if the antibody defect worsens or breakthrough infections occur despite prophylactic antibacterials.

## Bronchiectasis management to minimize infections

**Figure 1.**
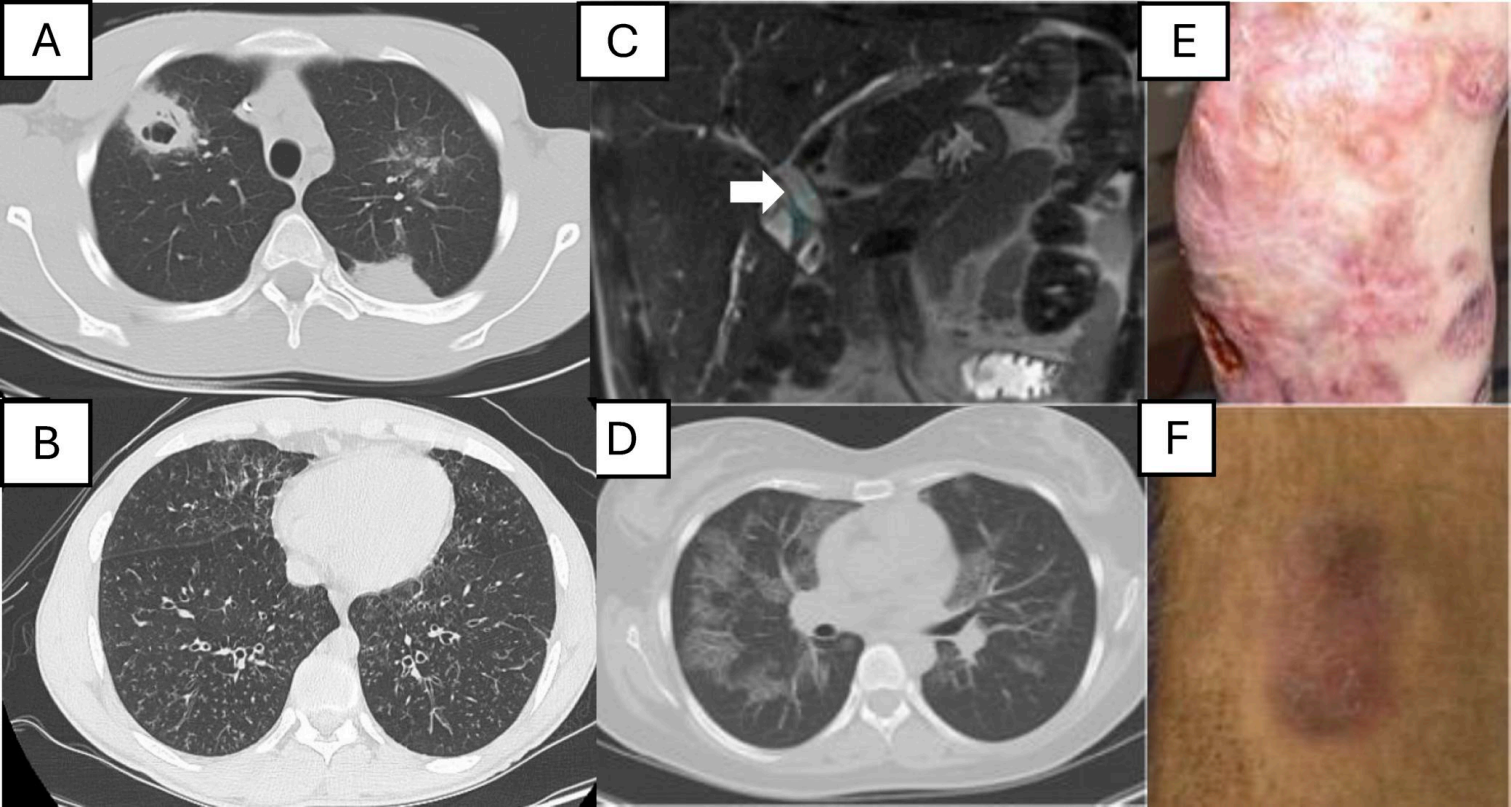
**Imaging and clinical findings of infections and inflammation in IEI. (A)** *Nocardia farcinica* pneumonia in a 17-year-old patient with CGD that did not improve until corticosteroids were added. **(B)** Tree-in-bud airway inflammation with pulmonary *P. aeruginosa* and NTM infection in a 21-year-old patient with DOCK8 deficiency. **(C)** Dilation of common biliary duct (arrow) on liver Magnetic resonance cholangiopancreatography (MRCP) with *Cryptosporidium* complicating DOCK8 deficiency. **(D)** Pulmonary alveolar proteinosis (PAP) found on chest imaging in a 22-year-old patient who presented with *Cryptococcus gattii* meningitis and then found to have neutralizing anti-GM-CSF antibodies. PAP can also be seen with GATA2 deficiency and other conditions. **(E)** Rubella associated skin granulomas on the lower extremity of a 37-year-old patient with TAP2 deficiency. **(F)***Helicobacter bilis*–associated lower extremity skin lesion in a 17-year-old patient with XLA.

Prevention of lung infection is dependent on both the hematopoietic compartment as well as mucociliary clearance. Bronchiectasis is the permanent dilation of the bronchi, resulting in impaired mucociliary clearance, leading frequently to thickened secretions and inability to effectively clear pathogens. For patients with IEI, particularly those with PADs, such as CVID and XLA, as well as CIDs, bronchiectasis often results from repeated pneumonias (18). In some IEI, such as STAT3-HIES, the remodeling of lung tissue is impaired and the mucociliary clearance abnormalities can be more pronounced (19, 20). Bronchiectatic airways typically become colonized or chronically infected with microbes, frequently Gram-positive and negative bacteria, such as *S. aureus* and *Pseudomonas aeruginosa*, respectively, and less frequently NTM and fungi, including molds (e.g., *Aspergillus*) (Fig. 1). These infections can be especially difficult in those with IEI in which both the impaired immune response and the impaired mucociliary clearance can result in particularly severe infections.

The focus of prevention of infection for those with bronchiectasis is aimed at improving the mucociliary clearance and diminishing the bacterial colonization of the airways. Due to the relative prevalence of cystic fibrosis (CF) compared to IEI associated bronchiectasis, many of the CF therapies have been adopted for non-CF bronchiectasis (21, 22). Airway clearance techniques include oscillatory positive-end-pressure devices and hypertonic saline nebs (typically 3 or 7% saline) given after albuterol to minimize bronchospasm. Percussive vests can be helpful, but we recommend avoiding for STAT3-HIES as rib fractures are particularly common. Some devices combine positive expiratory pressures, oscillation, and nebulization, which can be useful for people with complex airway anatomy, as seen in some cases of STAT3-HIES. As discussed above, azithromycin is frequently used for those with bronchiectasis. In cases of severe bronchiectasis/large airway malformations, we recommend mycobacteria sputum cultures (induced if feasible) prior to initiation of azithromycin prophylaxis, since NTM can quickly become resistant to azithromycin when used as a single agent. For young children with bronchiectasis that are unable to expectorate sputum for culture, we do not need feel a bronchoscopy for mycobacterial culture is needed unless there is clinical suspicion for NTM. Inhaled antibiotics, such as inhaled tobramycin given every other month, are frequently used to suppress *Pseudomonas* chronic infection for CF patients, and we support nebulized antibacterials if repeated infections continue despite airway clearance techniques and azithromycin (21, 23). Of note, tobramycin systemic absorption from nebulization can cause toxicity in patients with renal insufficiency. Antifungal nebulization, such as inhaled amphotericin or voriconazole, may be considered when there is chronic mold infection with bronchiectasis (24). Patients with STAT3-HIES are particularly prone to hemoptysis and should be counseled to stop inhaled medications (including hypertonic saline) and seek guidance if hemoptysis occurs.

## Immunosuppressive medications and infection concerns

Very limited evidence/guidance exists specific to the use of immunomodulatory medications in IEI, but they are increasingly used as precision medicine practices evolve. Immunosuppressive drugs are often given in combination or sequentially, leading to additive infection risks. We have highlighted several immune suppressant medications that we use most frequently but acknowledge this is far from a comprehensive list (25).

### Corticosteroids

Steroids generally have broad effects on multiple arms of the immune system, including phagocyte, T cell, and eosinophil function, leading to increased risk for multiple pathogens, including molds, PJP, herpesviruses, hepatitis viruses, and *Strongyloides* (26). We recommend considering PJP prophylaxis based on steroid dose, duration, and combination with other therapies; for instance, with the equivalent of 20 mg/day prednisone adult dosing for 4 wk. For IEI affecting T cell number/function, a lower threshold for PJP prophylaxis is prudent with corticosteroids. We monitor for herpes infections, but there is limited evidence for prophylaxis; we support using acyclovir or valacyclovir prophylaxis in patients who develop recurrent HSV infections or have had zoster reactivation. It is important to screen for *Strongyloides*, especially for those with high risk, and provide empiric treatment (e.g., ivermectin) to those with any lifetime exposure in an endemic area (tropical and subtropical areas across the globe).

### Rituximab

B cell depletion with rituximab is frequently used for IEI associated with autoimmunity. We support screening all patients for hepatitis B viral (HBV) infection (HBV surface antigen, HBV core antibody, and HBV DNA) before initiation, as B cell–depleting therapy has a high risk of hepatitis B reactivation (27). We have seen hepatitis B core antibody positivity in patients on IgRT without known hepatitis B risks, and this may represent false positivity (28). Because reactivation of hepatitis B can be fulminant and hepatitis B suppression medications have minimal risks, we typically consult hepatology and consider using a hepatitis B treatment, such as entecavir, if there is any possibility of hepatitis B infection. Rituximab also has a black box warning for progressive multifocal leukoencephalopathy (PML), and this risk should be considered prior to use in patients with other risks such as IEI with increased risk of PML (e.g., STAT1 gain-of-function [GOF], DOCK8 deficiency, and severe CD4 lymphopenia) (29). Finally, cases of PJP have been reported even in patients on rituximab monotherapy, and prophylaxis should be considered, particularly for those with CID (30).

### JAK inhibitors (JAKi)

JAKi are increasingly used to treat immune dysregulation in IEI, such as STAT1 and STAT3 GOF. Prior to use, patients should be screened for latent tuberculosis (TB) infection and hepatitis B and C, including through PCR techniques if antibody production is poor. In use, the most common concern is for herpes zoster reactivation, and patients with STAT1 and STAT3 GOF have increased risk for herpes viral infections without the added immune suppression (31). The recombinant subunit vaccine for herpes zoster can be given prior to initiation, but as these patients have variable vaccine responses, we and others recommend prophylaxis with acyclovir or valacyclovir. However, in an European Society for Immunodeficiencies study examining JAKi use for these diseases, antiviral prophylaxis was given to the minority of patients, and zoster was rare (32). We counsel patient about signs of herpetic disease to initiate therapy promptly. We use valganciclovir as secondary prophylaxis if the patient has a history of CMV disease; the use of letermovir in this setting is less clear. PJP is less of a concern but has been reported (32) and therefore may be considered for those with T lymphopenia. JAK inhibition can also lead to polyomavirus reactivation, such as BK and JC virus, and PML has been attributed to JAKi, which is especially concerning considering that STAT1 GOF also confers risk for PML (33). Unfortunately, JC viremia and viruria are not good predictors for PML risk, and whether JAK inhibition will increase (or decrease) the risk in those with STAT1 GOF is unknown. We recommend discussion of these risks and a low threshold for brain imaging and cerebrospinal fluid (CSF) analysis if there are neurologic concerns. Lastly, because inflammatory disease may worsen when doses are held, we typically continue JAKi through minor infections, such as upper respiratory tract infections.

### TNF-α inhibitors (TNFi)

Inflammatory bowel disease in CGD can be difficult to manage and TNFi may be considered; however, there have been reports of bacterial, fungal, or viral infections developing, supporting extra caution (e.g., increased prophylaxis, low suspicion for infections) or consideration of alternatives (34, 35).

### Tocilizumab

Prior to starting therapy, screening is recommended for latent TB. Although there is a black box warning for invasive fungal infections and opportunistic bacterial and viral infections, in our experience, the added infectious risks when using this agent are typically low, and additional prophylaxis is typically not recommended.

## Vaccines

While some IEI are defined by deficient specific antibody production, there are others in which close attention to vaccination is an important infection prevention intervention, e.g., pneumococcal and meningococcal vaccination for people with complement disorders. In addition, even if patients have antibody defects requiring IgRT, there are important vaccines that elicit a T cell response in addition to the B cell antibody response, including the influenza and SARS CoV2 vaccines (36, 37). These vaccines are updated each year based on circulating strains, and thus IgRT may not provide the most up to date protection due to the time lag between collection of serum from donors and administration the IgG product. Close contacts of people with IEI should also be vaccinated to minimize exposures. Adjuvanted recombinant subunit vaccine for herpes zoster is approved universally in adults aged 50 years and older and in adults aged 18 years and older who are or will be at increased risk of herpes zoster due to immunodeficiency or immunosuppression and so should be considered for many with CID or receiving immune suppressants (38). RSV vaccines are now approved for individuals aged 50–74 years with increased risk for severe disease and thus should be considered for those with CID and/or chronic lung disease (e.g., bronchiectasis).

There are some IEI in which live attenuated vaccines are contraindicated. Avoiding live viral vaccines in those with SCID is clear; however, in patients with CIDs who appear to tolerate live viral vaccines, there can be unusual manifestations such as vaccine-strain rubella granulomas (Fig. 1) (39, 40). Unfortunately, the diagnosis of CID may be made after the first rubella vaccine is given. For children in whom the tolerance of live viral vaccines is less clear, we recommend giving live viral varicella vaccine separately from and prior to Measles Mump Rubella (MMR) as vaccine-strain varicella can be treated with acyclovir. For patients on IgRT, there is decreased efficacy of attenuated live viral vaccines, and therefore should not be given. Oral bacterial vaccinations should be avoided in certain IEI (e.g., CID and neutrophil disorders). Specifically, inactivated *Salmonella typhi* vaccine should be given to people with IFNγ/IL-12 pathway disorders instead of oral live vaccine. Finally, Bacillus Calmette–Guérin (BCG) should not be given to those with SCID, CGD, and defects of the IL12/IFNγ/STAT1 pathway.

## Environmental considerations in infection prevention

### Avoiding molds

For IEIs with risk of mold infections, including CGD, STAT3-HIES, and severe chronic neutropenia, it is important to educate patients on avoidance of high mold exposure activities such as mulching, lawn mowing, and hayrides. Patient with CGD exposed to large amount of mold can develop fulminant pneumonitis, leading to respiratory failure. Treatment for “mulch pneumonitis” is corticosteroids in addition to antifungals (typically an azole), as well as broad-spectrum antibiotics given risk of polymicrobial infection (41).

### Water exposures

People with CGD are at risk for certain infections from brackish water, such as *Chromobacterium violaceum*, and should avoid swimming in non-chlorinated water (42). Patients with CID such as hyper IgM syndrome/CD40L deficiency and DOCK8 deficiency should be advised on safe water supplies and avoiding high-risk activities such as water parks due to the risk of *Cryptosporidium* (discussed more below) (43, 44), Finally, *Giardia* can cause chronic infection in patients with antibody deficiencies, and thus outdoor water exposures, such as creeks, should be avoided.

## Diagnostic challenges

In IEI, promptly and accurately diagnosing infections is essential, but this is not always straightforward. The following are some pearls from our experiences managing these patients.

### Atypical signs of infection

In conditions with impaired cytokine signaling, signs of inflammation, such as fever, redness, and pain, may be more subtle (for example, “cold abscesses” in STAT3-HIES), and inflammatory markers, such as C-reactive protein (CRP) and increased white blood cell counts, may appear misleadingly normal. In addition to decreased signs of inflammation, the formation of pus is impaired in leukocyte adhesion deficiency and neutropenia. Having a low threshold for imaging, obtaining sputum cultures when possible, and at times serum fungal antigen markers and cell-free DNA detection techniques can help direct diagnostic workup.

### Diagnosing lung infections

The dense parenchymal inflammation in CGD lung infection is very different than the more airway-based infections seen in purulent pneumonias or bronchiectasis exacerbations seen in patients with HIES or PADs. Therefore, diagnosis of lung infections in CGD is best made by tissue biopsy, ideally with interventional radiology; open lung biopsy can be considered if initial diagnostics are inadequate. For those with airway-based pneumonias, bronchoscopies with bronchioloalveolar lavage yield high results. Particularly in STAT3-HIES, lung biopsies should be avoided, if possible, due to the impaired wound healing and the higher risk of pneumothorax-associated persistent air leak (45).

### Fastidious bacteria

Close collaboration with specialized microbiology laboratories is necessary to allow detection of these organisms. *Granulibacter bethesdensis* is a Gram-negative bacillus that was first identified in patients with CGD in 2006 (46). Infections are often indolent and cause lymphadenitis and splenic lesions. *G. bethesdensis* is fastidious and requires special microbiology media. Interestingly, *G. bethesdensis* infections have not been reported with other IEI.

*Campylobacter* and *Helicobacter* species are facultative Gram-negative bacteria that are typically restricted to the gastrointestinal tract. However, in XLA, they can become disseminated and cause chronic bacteremia with recurrent fevers and skin and soft tissue infection (Fig. 1) (47, 48). It is necessary to have a low threshold of suspicion for these organisms in patients with XLA with rashes, especially boggy bruise-like changes over the shins. These bacteria are difficult to isolate and difficult to treat, and cell-free DNA techniques for microbiologic diagnosis can be useful to detect them. Therapy for *Campylobacter* and *Helicobacter* requires combination antimicrobials, usually with IV antibiotics. Clearance is difficult even after prolonged courses (i.e., months to years), and HSCT may be required for cure. *Mycoplasma* and *Ureaplasma* species can also cause infections in CVID and CIDs, including bone and joint disease (49). Because antibody-based diagnostics are typically unreliable in those with humoral immunodeficiencies, and *Mycoplasma*/*Ureaplasma* are difficult to isolate, diagnosis often relies on metagenomic next-generation sequencing (mNGS) from biopsied tissue and/or cell-free DNA techniques.

### Chronic viral infections

#### Enterovirus

Chronic enterovirus meningoencephalitis has been long recognized in XLA, with high morbidity and mortality (50). It is essential to maintain high suspicion and consider CSF studies and potentially a brain biopsy to diagnose this promptly. Treatments have included high-dose IVIG as well as intraventricular IgG, but responses remain poor. Antivirals against enterovirus are in development and could potentially be obtained (50, 51). We support consideration of HSCT if diagnosed early to prevent progression. Although the live poliovirus vaccine is no longer routinely given in the U.S., it is still used globally for disease outbreak control and can cause vaccine-associated paralytic poliovirus in those with XLA and other IEI.

#### Norovirus

Chronic norovirus has been recognized in patients with primarily CVID and CIDs, leading to malnutrition and poor quality of life (52). Many treatments have been tried, including nitazoxanide, ribavirin, and oral IgG, without clear success, and viral-specific T cell therapies are being studied (53). In some cases, treating the immune dysregulation related to the enteropathy, (e.g., with sirolimus) can have positive outcomes raising the question of the contributing roles of the virus and the dysregulated immune response.

#### Rubella

Rubella, as either vaccine strain or wild type, can cause skin granulomas and, at times, systemic disease in patients with CIDs, but also in those with antibody deficiencies (Fig. 1) (39, 40). Diagnosis has relied on stains of affected tissues as well as PCR. Similar to chronic norovirus, many treatments have been tried (e.g., nitazoxanide) without consistent success, except for resolution with HSCT (54). Also, like chronic norovirus, in the absence of specific antiviral therapy, it remains unclear whether the target of therapy should be dampening the abnormal immune response or increasing the antiviral immunity, as both approaches have had partial response (55).

#### Aichi virus

Aichi virus is an RNA virus in the *Picornaviridae* family that has recently been described as causing chronic infection in patients with XLA and other PADs/CIDs (56, 57). Aichi virus appears to cause kidney and liver disease, with the full extent of pathology still being defined. Diagnosis has been with mNGS, with techniques for identification, proof of causality, and therapy still being defined. Due to the severity of illness potentially from chronic infection, early HSCT may be considered (58).

For other examples of chronic viral infections in IEI, we recommend this review, which discusses the examples above as well as measles, human papillomavirus, and parvovirus B19 (59).

##### Endemic dimorphic fungi

*Coccidioides* is a dimorphic fungus prevalent in the soil in the Southwestern U.S. and South America. *Coccidioides* typically causes a self-limited pulmonary infection. However, in certain IEI, disseminated disease can occur, typically to the axial skeleton and/or brain. IEI associated with *Coccidioides* risk include STAT3-HIES, STAT1 GOF, and defects of the IL12/IFNγ pathway (60, 61, 62). Immunosuppressive medications, such as TNFi and JAKi, can also lead to disseminated coccidioidomycosis (60, 63). It is essential to be aware of those at risk for disseminated *Coccidioides* to quickly diagnose and treat the infection. Diagnosis is typically by antigen and/or antibody assays (if the patient can make specific antibodies); culture is also possible, but fungal growth can aerosolize and be toxic, and microbiology laboratories should be warned of the possibility of growth. Treatment of disseminated disease can be difficult—central nervous system (CNS) disease can only be suppressed, not eradicated—so patients should remain on suppressive antifungals indefinitely. Due to the difficulty in treating disseminated *Coccidioides*, prophylactic fluconazole should be offered for any patient with at-risk IEI living or traveling to endemic regions—noting that the geographical areas at risk for *Coccidioides* are evolving over time.

*Histoplasma* can also cause significant disseminated disease in certain IEI such as STAT3-HIES and IFNγ/STAT1 pathway defects. Of note, disseminated intestinal histoplasmosis can present in STAT3-HIES in a subacute manner mimicking inflammatory bowel disease (64). In addition, *Histoplasma* can lead to hemophagocytic lymphohistiocytosis with a high infectious load, including in STAT3-HIES. Diagnosis of disseminated disease is by *Histoplasma* blood or urine antigen. The 2025 Infectious Diseases Society of America Clinical Practice Guideline suggests a framework for considering degree of immunocompromise, including IEI, in decisions regarding whether to provide treatment for asymptomatic, previously untreated *Histoplasma* pulmonary nodules (65).

##### Parasites

*Cryptosporidium* is a parasite that causes self-limited diarrhea in immunocompetent individuals. In some CIDs, *Cryptosporidium* causes a chronic infection, primarily of the small bowel. Although this can result in chronic diarrhea, the more difficult complication is biliary disease, which can progress to biliary cirrhosis (Fig. 1). Because diarrhea can resolve during biliary disease, one must have a low suspicion to investigate. The IEI in which this is reported most frequently are CD40 ligand deficiency, DOCK8 deficiency, MHCII deficiency, and IL-21 receptor deficiency (43, 44, 66). The concentration of parasites in the stool may be low, making typical microbiology stains less sensitive; PCR techniques, typically done as part of a gastrointestinal pathogen panel, are more sensitive. Treatment relies on immune reconstitution, typically with HSCT, ideally prior to the development of significant liver disease. Combination liver/marrow transplants have been performed in some patients with mixed results (67, 68). Although anti-parasitic agents are not very effective in clearing this organism, we suggest treating with nitazoxanide with or without azithromycin to minimize bowel disease, including through HSCT.

##### Mycobacteria

There is significant clinical variability in the group of IEI referred to as Mendelian susceptibility to mycobacteria (MSMD). While some require HSCT (e.g., recessive complete IFNγR1 or R2 deficiency), in other milder forms (e.g., dominant IFNγR1 deficiency and dominant negative STAT1 deficiency), prophylaxis with azithromycin is typically sufficient after NTM disease is controlled. Prophylaxis against NTM is extrapolated from data acquired in HIV studies; however, although the weekly prophylaxis dose used in HIV is likely adequate, we usually dose the azithromycin daily at 5 mg/kg/day for children up to 250 mg daily for adults (69). We also recommend anti-mycobacterial prophylaxis with azithromycin for infants with complete athymia until thymic implantation is performed (70). BCG vaccine given prior to diagnosis with SCID is a rare situation in which prophylaxis with isoniazid and rifampin could be considered though standard recommendations do not exist (71). Lastly, we do not recommend IFNγ as prophylaxis for MSMD; however, IFNγ therapy can be a useful adjunct to anti-mycobacterial antibiotics to treat disseminated NTM in certain IEI such as NEMO, IκBα deficiency, IL12RB1 deficiency, and autosomal dominant IFNγR1 deficiency.

##### IEI mimickers

There is increasing recognition of anti-cytokine antibodies and their role in infection susceptibility (72). Compared to most IEI in which infections usually start in childhood, these typically present in adults with opportunistic or severe infections. *Nocardia* and *Cryptococcus* CNS infection should raise consideration for anti-GM-CSF antibodies; of note, the infection can precede the classic presentation of anti-GM-CSF as pulmonary alveolar proteinosis (Fig. 1) (73). Disseminated NTM in adults should raise concern for anti-IFNγ antibodies. In the U.S., anti-IFNγ antibodies are typically found in East Asian women. In East Asia, the gender difference is less pronounced (74). Severe viral illness, such as severe COVID, neuroinvasive West Nile disease, and yellow fever vaccine disease has been seen in those with anti-IFNα antibodies (75, 76, 77). Thymomas usually present in adulthood and can be associated with hypogammaglobulinemia (Good’s syndrome) with associated infection and/or with infections related to autoantibodies (78).

## Management challenges and quirks

The many management challenges that arise in treating infections in IEI is beyond the scope of one review, but below are bits of information that are helpful while caring for these patients.

### Exuberant inflammation in CGD

Due to the exuberant but ineffective inflammatory response, some infections are best treated with a combination of culture-guided antimicrobials plus corticosteroids with a slow taper; these infections include *S. aureus* liver abscesses and *Nocardia* pneumonia (Fig. 1) (79, 80). In general, we recommend avoiding IFNγ while treating infection in CGD to allow better monitoring of fever and laboratory trends.

### Medication quirks and adverse events

Every medication has the possibility of adverse events, and many have possible drug–drug interactions. We will not review the risks of every antimicrobial but highlight some of the less frequently recognized but important associations that we have seen.

#### TMP/SMX

It is essential to consider the purpose of prophylactic TMP/SMX when dosing. While 3 days/wk is adequate for the prevention of PJP in patients with SCID and some other CIDs, it is inadequate to prevent bacterial infections such as *S. aureus*. Therefore, dosing for prophylaxis in CGD and HIES should be twice daily every day. In addition, we support the use of folinic acid in infants with SCID to minimize TMP/SMX-induced neutropenia (2).

#### Azoles

Azole antifungals are metabolized by the CYP450 enzymes. Therefore, azoles can diminish the metabolism of corticosteroids, and there the patients may “see” a much higher dose of steroids than expected when on azoles. This can lead to adrenal insufficiency when otherwise not suspected, including, at times, with inhaled/intranasal corticosteroids (81). Voriconazole is associated with photosensitivity and, over time, increased incidence of skin cancers (82). When a mold-active antifungal is required for long-term therapy or prophylaxis, an alternative to voriconazole should be considered if sun exposure will occur. Retinal exams are indicated for patients on long-term voriconazole given reported retinal toxicity and optic neuropathy (83). In addition, long-term use of voriconazole, especially at higher drug levels, can result in fluorosis that may manifest as periostitis (84). Posaconazole can be associated with pseudohyperaldosteronism, leading to hypertension, hypokalemia, and signs of heart failure (85). Sometimes this can be ameliorated with lowering the dose or with use of spironolactone.

#### QTc prolonging drugs

Many of the antibiotics used in IEI, such as azithromycin, fluoroquinolones, and azoles (e.g., itraconazole and posaconazole), can increase the QTc interval. As these medications may be used in combination, it is prudent to have a baseline and on-therapy EKG for QTc while using multiple QTc prolonging agents.

### Therapeutic drug monitoring

One of the great challenges in IEI antimicrobial prophylaxis is medication adherence, which can not only leave the patient at risk for infection, but can also potentially increase antimicrobial resistance. We frequently obtain serum drug levels when this is a concern and is most easily done with sulfa levels for those on TMP/SMX and antifungal levels for those on triazoles (e.g., voriconazole, posaconazole, and isavuconazole).

### Women’s health and pregnancy

As patients with IEI are living longer with improved diagnostics and therapies, women’s health concerns, including pregnancy and pregnancy prevention, are becoming more pertinent. Regarding pregnancy prevention, in STAT3-HIES, we had met some concerns about infection risks with intrauterine devices (IUDs). We have now followed many women with STAT3-HIES with IUDs without increased infection risk, and IUDs may be preferable to oral contraceptives due to concerns of hypercoagulability with STAT3-HIES–associated vasculopathy (86). Antimicrobial prophylaxis during pregnancy can be challenging, as many medications used are contraindicated during pregnancy. Specifically, TMP/SMX should be avoided, especially during the first trimester and then again close to delivery due to potential risks of jaundice and hemolytic anemia. Chronic azole use has also been associated with congenital birth defects. Azithromycin and β-lactams are safe in pregnancy, and topical antifungals can be used for mucocutaneous *Candida* infections, but the patients should be counseled for gaps in coverage and to seek care if there is concern of infection for prompt diagnosis and treatment. In addition to discussions around pregnancy and family planning, certain IEIs predispose to Human Papillomavirus (HPV) infection and increased rates of HPV-related malignancy (i.e., vulvar and cervical cancer), such as GATA2 deficiency and DOCK8 deficiency. Consideration and screening for HPV-related malignancies is necessary. We also support HPV vaccination for the patient if curative HSCT is not soon. It should be noted, however, that the efficacy of HPV vaccination in prevention of these malignancies is unclear because the HPV serotypes causing malignancy may differ and the efficacy of the vaccine may be less.

#### Perioperative care

The need for surgery with IEI can cause anxiety both for the risk of infection but also due to potentially abnormal healing. It is important for the IEI focused physician and the surgeon to have open communication to minimize these risks. For patients with increased risk of skin and soft tissue infections, we recommend efforts to decrease *S. aureus* colonization preoperatively as well as peri-surgical anti-*Staphylococcal *treatment. Decolonization can be with increased use of antiseptics such as chlorhexidine, dilute bleach baths/swimming in chlorinated pools, and intranasal mupirocin. When hardware is placed, such as in orthopedic surgeries, we tend to increase the time of perioperative antibiotics; for instance, in STAT3-HIES, where these surgeries occur relatively commonly, we recommend anti-*Staphylococcal *antibiotics (such as linezolid due to methicillin-resistant *S. aureus* coverage and excellent tissue penetration) starting 1–2 days preoperatively and continuing for 7–10 days postoperatively while the incision heals. For patients with bronchiectasis, airway clearance should be stressed, and it is prudent to have sputum cultures in advance of surgery to clarify the current airway colonizing microbes in case of bronchiectasis exacerbation. Healing can be abnormal in certain IEI; for instance, in CGD, there can be an exuberant inflammatory response at the surgical incision that can lead to wound dehiscence but improves with corticosteroids. In STAT3-HIES, healing after lung surgeries is often impaired with prolonged bronchopleural fistulae (45).

## Future directions

Greater genetic diagnosis and understanding of the pathogenesis of IEI along with increasing development of immune modulatory medications is changing the approach to comprehensive management of patients with IEI. Specifically, there is an evolution from primarily supportive measures frequently dominated by antimicrobial prophylaxis and/or IgRT toward precision medicine to treat immune dysregulation through small molecules and monoclonal antibodies. For instance, the use of CXCR4 antagonists, such as plerixafor and mavorixafor, to treat warts, hypogammaglobulinemia, infections, myelokathexis syndrome caused by GOF CXCR4 mutations results in significantly less wart and infection burden (87, 88). However, with some of these changes to therapy, we will likely encounter a different set of infectious complications related to immune modulatory medications that will require future study. In addition, our methods to diagnose fastidious and viral infections are improving through cell-free DNA-based techniques and metagenomic studies leading to the discovery of more pathogens for which patients with IEI have increased risk. Finally, approaches to HSCT and gene therapy/editing are changing with improved outcomes and thus more willingness to “cure” more complicated patients who may have a significant infection burden. In sum, the close partnership between infectious diseases physicians and immunologists will continue to be essential for preventing, diagnosing, and treating infections as part of the comprehensive care for patients with IEI.

## Data Availability

No new data were generated or analyzed in support of this article.
